# Effects of PICS bags on insect pests of sorghum during long-term storage in Burkina Faso

**DOI:** 10.1016/j.jspr.2019.07.010

**Published:** 2019-09

**Authors:** Antoine Waongo, Fousséni Traore, Malick N. Ba, Clémentine Dabire-Binso, Larry L. Murdock, Dieudonné Baributsa, Antoine Sanon

**Affiliations:** a Laboratoire Central d’Entomologie Agricole de Kamboinsé (LCEA-K), Institut de l’Environnement et de Recherches Agricoles (INERA), 01 BP 476 Ouagadougou 01, Burkina Faso; b International Crops Research Institute for the Semi-Arid Tropics, Niamey, Niger; c Department of Entomology, Purdue University, 901 West State Street, West Lafayette, IN, 47907, USA; d Laboratoire d’Entomologie Fondamentale et Appliquée, UFR/SVT, Université Ouaga I Pr Joseph Ki-Zerbo, Burkina Faso

**Keywords:** PICS bags, Sorghum, *Rhyzopertha dominica*, Long term storage, Resurgence

## Abstract

The PICS bags, originally developed for cowpea storage, were evaluated for sorghum (Sorghum bicolor) preservation. Batches of 25 kg of sorghum grain were stored in 50 kg PICS or polypropylene (PP) bags under ambient conditions for 12 months and assessed for the presence of insect pests and their damage, seed viability and, oxygen and carbon dioxide variations. The grain was incubated for 35 days to assess whether any insects would emerge. After six months of storage, oxygen levels decreased in the PICS bags compared to polypropylene bags. After 12 months of storage, only two pests, *Rhyzopertha dominica* and *Sitophilus zeamais* were found in the PICS bags. However, in PP bags there were additional pests including *Tribolium castaneum* and *Oryzeaphilus mercator* and *Xylocoris flavipes*. Grain weight loss and damage caused by these insects in the PP bags were significantly higher compared to those stored in PICS bags. Germination rates of sorghum grains stored in PP bags decreased significantly while no changes were observed in grains stored in PICS bags when compared to the initial germination. After the incubation post storage period, there was a resurgence of R. dominica in sorghum grains from PICS bags but the population levels were significantly lower compared to polypropylene bags. PICS bags preserved the quality and viability of stored sorghum grains and protected it from key insect pests. The PICS technology is effective for long-term sorghum storage but the potential resurgence of insects in low-oxygen environment calls for further research.

## Highlights

• PICS bags can use for the storage of sorghum grains over a 12-months period.
• After 12 months of storage, *R. dominica* is the only species that emerged from sorghum grains stored in the PICS bags.
• *R. dominica* would be less sensitive to lower O_2_ concentrations in the storage environment.

## 1 Introduction

Agriculture in the Sahel is dominated by the production of traditional grains such as millet [*Pennisetum glaucum* (L.)] and sorghum [*Sorghum bicolor* (L.) Moench] (World Bank, 2011). In Burkina Faso, sorghum is the predominant cereal. In 2015, Burkina Faso produced 1,707,613 tons on 1,548,404 ha; representing 38.21% of total cereal production and 42% of the total cereal production area (DGESS, 2015). In addition to its high nutritional value, sorghum grain is also a source of diverse compounds including tannins, phenolic acids, anthocyanins, phytosterols and policosanols (Dykes and Rooney, 2006; Dlamini et al., 2007).

Traditionally, sorghum is stored as panicles in straw or mud granaries (Waongo et al., 2013). In traditional storage conditions, in Burkina Faso, sorghum grains are attacked by several insect pests, with the lesser grain borer *Rhyzopertha dominica* (F.) (Coleoptera: Bostrychidae) being the most important (Waongo et al., 2015). Infestation of stored grains by *R. dominica* is known to cause losses in both quality (Williams et al., 1981) and quantity (Brower and Tilton, 1973). In Burkina Faso, losses during sorghum storage are estimated to be 6.8% (Loada et al., 2015). Damage to seed and residue produced insect feeding reduce grain quality and decreases the essential amino acid contents (Jood et al., 1995). Damage caused by *R. dominica* also reduce seed viability and seedling vigor (Jilani et al., 1989). Improved storage is needed because traditional storage is largely ineffective.

Hermetic storage of grain using Purdue Improved Crop Storage (PICS) bags represents a promising way of post-harvest grain storage. This technology has the advantage of being easy to use, affordable, and does not require the use of chemicals that may be harmful to human health (references). The effectiveness of PICS bags for controlling storage insect pests has been demonstrated on different legume crops including cowpea, bambara groundnut, groundnut, pigeonpea, mungbean and common bean (Sanon et al., 2011; Baoua et al., 2012, 2014; Affognon et al., 2014; Baoua et al., 2014a; Mutungi et al., 2015; Sudini et al., 2015). Likewise, PICS bags are effective in protecting cereal grain against different insect species feeding on rice, maize, wheat, sorghum, cassava, and *Hibiscus* seeds (Baoua et al., 2014b; Njoroge et al., 2014; Hell et al., 2014; Martin et al., 2015; Amadou et al., 2016; Baoua et al., 2016b; Williams et al., 2017a, b).

A recent study by Williams et al. (2017a) focused on the effectiveness of PICS bags for preservation of sorghum seeds viability but they did not investigate the preservation from insect pest damage. Moreover, they only covered six months’ storage period while the average storage time of sorghum grains in Burkina Faso extend to nine months (Waongo et al., 2013). Therefore, the present study assessed the effectiveness of PICS bags for long-term storage of sorghum grains infested by insect pest over a period of 12 months.

## 2 Material and methods

### 2.1 Source of sorghum grains, packaging and storage conditions

Naturally infested sorghum grain (150 kg) was purchased from a local market (Sankaryare) in Ouagadougou (12°21′58″ North; 1°31′05″ West), Burkina Faso. The grain was divided into 6 batches of 25 kg and transferred into two types of 50 kg capacity bags: Treatment 1- control polypropylene (PP) bag which were standard woven bags of 25 mm thick; Treatment 2: PICS bag made of two liners fitted inside a woven bag. The PICS bags were closed tightly in accordance with the method described by Baributsa et al. (2010, 2013, 2015). All bags of both treatments were tightly sealed with a rubber cord. The bags were kept on pallets for a period of 12 months (March 2016 to February 2017) in a room with an average temperature of 29.29 ± 2.68 °C and relative humidity of 53 ± 20%. Rodent traps were placed in the room and bags were checked each week to ensure no damage.

### 2.2 Monitoring of oxygen and carbon dioxide concentrations

During the first eight months of storage, oxygen (O_2_) and carbon dioxide (CO_2_) levels in the bags were measured using a Mocon PAC Check^®^ Model 325 Headspace analyzer (Mocon, Minneapolis, MN, USA) at 12:00 local time.

### 2.3 Evaluation of insect infestation, grain damage and loss, and grain viability

Before tying the bags filled with sorghum, three samples of 500 g were collected randomly from each of the six bags to assess the initial insect infestation level, the number of grains with holes, and the initial weight of grains. Germination tests were carried out with four subsamples of 100 grains randomly collected from each 500 g sample. The same parameters were determined again at the end of the 12-months storage period. The infestation was assessed by counting the number of insects in the grain sample after sieving the grain through a 3 mm mesh sieve. Insects collected were sorted and counted according to their species to determine their abundance. However, grain damage was assessed from a subsample of 1000 grains from which we counted the number of damaged (with holes) grains; and the weight of damaged and undamaged grains. The percentage grain weight loss was calculated using the following formula (Boxall, 1986; Alonso-Amelot and Avila-Núñez, 2011):

$$Weight\;loss\;\left(\%\right)=\left[\frac{\left(a\;\times d\right)-\left(c\times b\right)}{a\;\times \left(d+b\right)}\;\right]\;\times 100$$

with: **a** = Dry weight of undamaged grains, b = number of undamaged grains, **c** = Dry weight of damaged grains and **d** = number of damaged grains.

### 2.4 Re-emergence of insects after storage time

Resurgence of insects was determined only after 12-months storage period. After the determination of the parameters aforementioned, each sample of 500 g was placed in a plastic jar of 1-L capacity covered with a mosquito net, and observed for 35 days. At the end of the observation period, the samples were sifted and the insects counted and grouped according to species.

### 2.5 Data analysis

The verification of the data distribution of each measured variables was performed using Shapiro - Wilk test. Data on number of insect pests after 12-months storage of sorghum grains were separated with the student's t-test. For other variables, when data were found normally distributed, we performed a Linear Model Analysis of Variance (ANOVA). When the p-value was significant, means comparisons were done using the Tukey's test. In contrast, when the data were not normally distributed, an analysis of variance following the model of Kruskal-Wallis. In this case, when the p-value was significant, means comparisons were made using the Dunn's test. All tests were performed with R software version 3.4.3 (2017-11-30) at the probability level of 5%.

## 3 Results

### 3.1 Oxygen and carbon dioxide concentrations

**Fig. 1:**
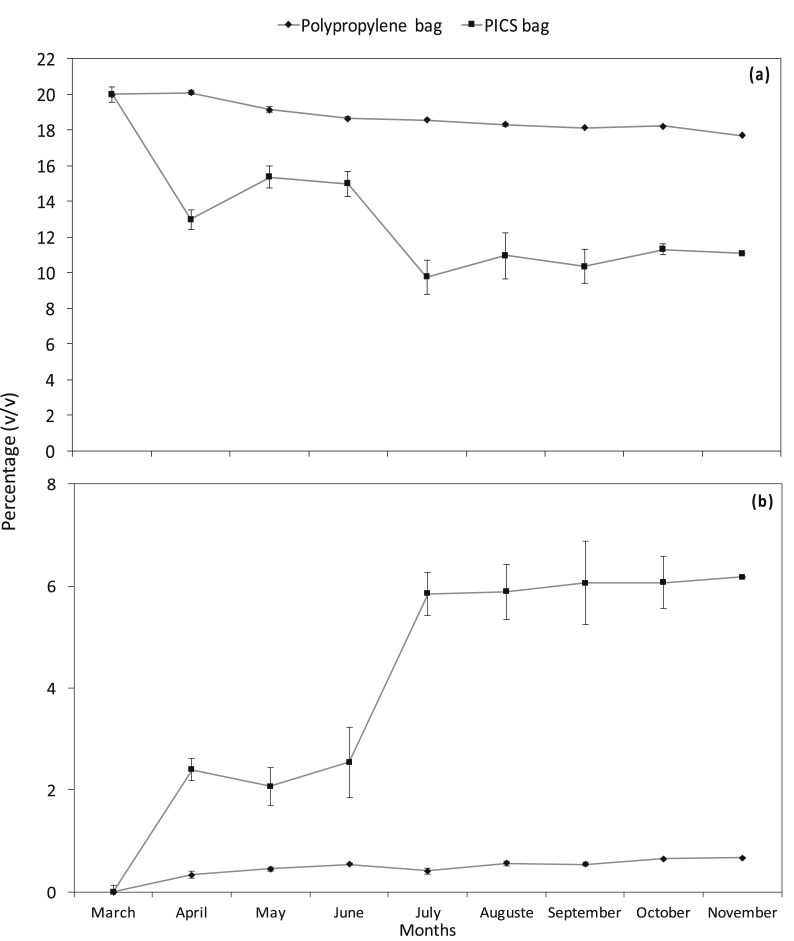
Monthly oxygen (a) (% Means ± S.E.) and carbon dioxide (b) concentration (%Means ± S.E.) in PICS and polypropylene bags over an 8-months period of sorghum storage.

Over the months, the O_2_ concentration decreased steadily in the PICS bags while the CO_2_ concentration increased. By the end of the 8-months period, the O_2_ concentration in the PICS bags decreased by about 8% compared with a 2% decrease in the PP bags (Fig. 1a). In the meantime the CO_2_ concentration increased by more than 6% in PICS bags compared to only 0.7% increase in the PP bags (Fig. 1b).

### 3.2 Insect infestation of sorghum grains

At the beginning of the storage period, four insect species were identified in the grains, namely *R. dominica*, *Sitophilus zeamais* Motschulsky (Coleoptera: Curculionidae), *Tribolium castateum* Herbst. (Coleoptera: Tenebrionidae) and *Oryzeaphilus mercator* Fauvel (Coleoptera: Silvanidae) (Table 1). At the end of the 12-months storage, only two insect species, *R. dominica* and *S. zeamais* were present in the PICS bags. In the PP bags, all four species were still present in addition to a predator species, *Xylocoris flavipes* (Reuter) (Hemiptera: Anthocoridae) not found at the outset of the experiment (Table 1).

**Table 1:** Number of insect pests of each species in 500 g samples at the beginning of the storage period and after 12-months storage in PICS and polypropylene bags.

Species	Initial number of insects (Means ± S.E.)	Number of insects after 12-months (Means ± S.E.)		Statistics
		PICS bags	Polypropylene bags	
***Rhyzopertha dominica***	2.33 ± 0.73b	12.67 ± 5.70b	187.67 ± 14.11a	χ^2^ = 7.2, Df = 2, P = 0.027
***Sitophilus zeamais***	2.66 ± 0.33b	2.33 ± 0.90b	5.0 ± 0.0a	F_2,6_ = 7.125, P = 0.026
***Tribolium castateum***	0.17 ± 0.16b	0.0 ± 0.0b	13.0 ± 4.0a	χ^2^ = 7.7143, df = 2, P = 0.021
***Oryzeaphilus mercator***	0.17 ± 0.17b	0.0 ± 0.0b	2.0 ± 1.0a	χ^2^ = 6.7879, df = 2, P = 0.033
***Xylocoris flavipes***	0.0 ± 0.0b	0.0 ± 0.0b	5.67 ± 3.71a	χ^2^ = 7.6235, df = 2, P = 0.022

Within a row, means followed by different letters were significantly different (Dunn test or Tukey test, α = 0.05).

### 3.3 Levels of insect pests after 12-months storage of sorghum grains in PICS and PP bags

After 12 months of storage, *R. dominica* was the only species that emerged from sorghum grains stored in the PICS bags but with a population 15 times less than that observed in the PP bags (Table 2). In addition to *R. dominica, T. castaneum, O. mercator, X. flavipes* and *Corcyra cephalonica* Stainton (Lepidoptera: Pyralidae) emerged from grain stored in the PP bags but not from grain held in PICS bags. (Table 2).

**Table 2:** Number of insect pests of each species in 500 g samples after a 12-months storage period in PICS and polypropylene bags.

Species	Number of insect after 12-months (Means ± S.E.)		Statistics
	PICS bags	Polypropylene bags	
***Rhyzopertha dominica***	12.67 ± 6.01b	147.67 ± 47.70a	t_1,4_ = 2.808, P = 0.048
***Tribolium castaneum***	0.0 ± 0.0b	6.0 ± 2.08a	t_1,4_ = 2.882, P = 0.045
***Oryzeaphilus mercator***	0.0 ± 0.0a	1.0 ± 1.0a	t_1,4_ = 1, P = 0.374
***Xylocoris flavipes***	0.0 ± 0.0b	8.33 ± 1.85a	t_1,4_ = 4.490, P = 0.011
***Corcyra cephalonica***	0.0 ± 0.0b	29.33 ± 7.21a	t_1,4_ = 4.064, P = 0.015

Within a row, means followed by different letters were significantly different (independent *t*-test, α = 0.05).

### 3.4 Grain damage and weight loss of sorghum stored for 12 months in PP and PICS bags

The percentage of damaged grains after 12-months of storage was nearly 30 times higher in polypropylene bags than in the PICS bags. In the PICS bags the final grain weight did not differ significantly from the initial weight (Table 3). Weight loss in the PP bags was fourteen times higher than that observed in the PICS bags (Table 3).

**Table 3:** Perforated grain and weight losses of grains stored in PICS bags and polypropylene bags over a 12-months storage period.

Treatments	Grains with holes (%Means ± S.E.)	Grain weight loss (%Means ± S.E.)
Initial level	0.50 ± 0.10b	0.08 ± 0.05b
PICS bags	0.57 ± 0.15b	0.35 ± 0.17b
Polypropylene bags	16.83 ± 5.32a	4.25 ± 1.26a
Statistics	F_2,6_ = 28.18; P = 0.0009	F_2,6_ = 30.45; P = 0.0007

Means bearing different letters within a column were significantly different (Tukey test, α = 0.05).

### 3.5 Seed viability after storage of sorghum grain in PP and PICS bags

**Fig. 2:**
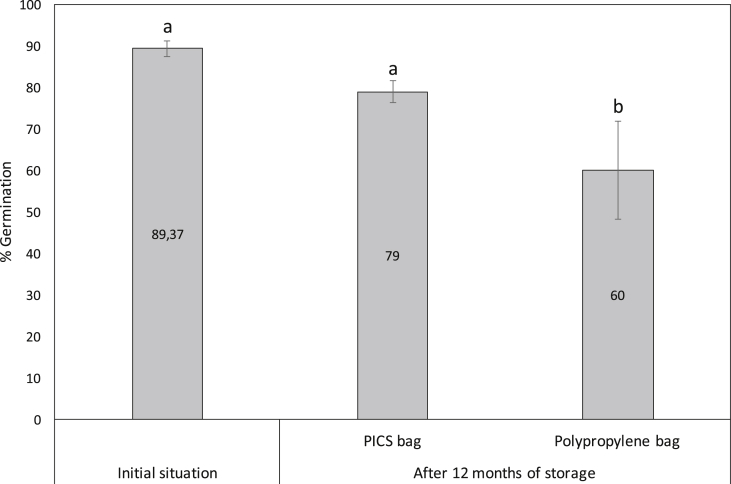
Mean germination (±S.E.) of sorghum grains stored in PICS bags and polypropylene bags over a 12-month period (column bearing different letters were significantly different (ANOVA Linear Model test, α = 0.05).

Sorghum grains stored in the PICS bags for 12 months germinated as well as it did at the start of the experiment. While the germination rate was slightly lower in PICS bags, the difference was not statistically significant. By contrast, germination decreased significantly when the grain was stored in PP bags (F_2,9_ = 17.85, P = 0.0007, Fig. 2).

## 4 Discussion

The present results revealed changes in the concentration of CO_2_ and O_2_ during the storage period for both PICS bags and PP bags. But in PP bags, which are permeable to air, only slight variation in O_2_/CO_2_ concentration was observed. However, in the confined atmosphere of the PICS bags, there was a significant decrease in the O_2_ content and a simultaneous increase in CO_2_ concentration. Similar trends in gas concentrations have been reported for PICS bags by several authors (Mutungi et al., 2015; Amadou et al., 2016). However, in the present study, the O_2_ and CO_2_ concentration decreased by 8% and increased by 6% respectively. Amadou et al. (2016) observed a decrease of 17% and an increase of 8%, respectively. Mutungi et al. (2015) observed a decrease in O_2_ and an increase in CO_2_ of about 15%. These differences in the variation in gases may be explained by the species present and numbers of insects present in the confined atmosphere of the PICS bags. In fact, in the present studies we observed four insect species initially present in the PICS bags while some studies observed only one insect species. According to Murdock et al. (2012), the respiratory activity of insects present in the confined atmosphere of the PICS bags, through inhalation of O_2_ and expiration of CO_2_, may explain the variation of respiratory gases in the storage bags.

Sorghum used in this experiment (purchased in a local market) was infested with four insect species including *R. dominica, S. zeamais, T. castaneum* and *O. mercator*. These species have been observed in traditional granaries in the Sudan ecological zone of Burkina Faso on sorghum (Waongo et al., 2013, 2015). *Sitophilus* sp have also been reported on stored maize in the same agroecology (Sudan ecological zone) (Baoua et al., 2016a). At the end of the 12-month storage period, some species, such as *X. flavipes* and *C. cephalonica,* not observed at the beginning of the study, were recorded only in the polypropylene bags. This may indicate that these species were initially present in the sorghum stocks either as eggs or in the larval stage and were not detected.

Incubation of grain for 35 days after the 12-months storage period showed that only a low resurgence of *R. dominica*. However, the rise in the number of *R. dominica* in the PICS bags was markedly lower than that observed in the polypropylene bags. These results may indicate that *R. dominica* is less sensitive to lower O_2_ concentrations in the storage environment compared with other species (*S. zeamais, T. castaneum, O. mercator, X. flavipes* and *C. cephalonica*), all of which had died off by the end of the 12-month storage period. As reported by Donahaye (1992) insects can develop resistance to low O_2_ environment. Mbata and Phillips (2001) showed that the eggs of *T. castaneum*, *P. interpunctella* (Hübner) and *R. dominica* are more tolerant to lower oxygen levels following a decrease in pressure. According to these authors, the immature stages of *R. dominica* were more tolerant to the lower pressure than the immature stages of the other two species. Some authors have shown that *R. dominica* eggs and adults are more tolerant to the lower oxygen levels than *T. castaneum* (Calderon and Navarro, 1980, Annis and Dowsett, 1993). Cheng et al. (2012) also showed that the 3rd and 4th instar larvae of *C. maculatus* are less sensitive to low oxygen levels compared to other stages of development. The ability in the insect to tolerate low oxygen is explained by a cessation of metabolic activities coupled with an increase in the mechanism of stress tolerance.

After 12-months of storage, substantial increases in the numbers of insect pests was observed only in the polypropylene bags, whereas in the PICS bags only two primary pest species *R. dominica* and *S. zeamais* were present but at population levels similar to those recorded prior to storage. PICS bags thus substantially suppressed the proliferation of *R. dominica* and *S. zeamais* and resulted in the death of secondary pests such as *T. castaneum*, *O. mercator* and the predator species *X. flavipes*. Murdock et al. (2012) noted that a reduction in O_2_ content in the PICS bags would result in a decrease in insect feeding activity and, consequently, of damage to grains. Our results showed that in addition to preventing or reducing the proliferation of insects, PICS bags preserve the quality of stored sorghum grains. After 12-months of storage, grain damage (with holes) and weight loss in PP bags were about 16% and 4%, respectively, while in PICS bags these variables did not differ significantly from those recorded at the beginning of the experiment. The current results corroborate with previous findings that PICS bags can maintain grain quality of several commodities over a period of 6–7 months (Sanon et al., 2011; Baoua et al., 2014a, Baoua et al., 2014b; Martin et al., 2015; Amadou et al., 2016; Williams et al., 2017a). The presence of secondary pests such as *T. castateum, O. mercator* and the predator insect species *X. flavipes* only in polypropylene bags is consistent with the degree of grain degradation observed (Delobel and Tran, 1993; Mukherjee and Nandi, 1993; Nansen et al., 2009; Yamada et al., 2013; Schöller and Prozell, 2014). In addition, PICS bags maintained the viability of the sorghum seeds over a period of 12-months. To the best of our knowledge the present work is the first to report that PICS bags can be used for up to 12-months for storage of seeds without significantly reducing seed viability. The present study, while demonstrating the effectiveness of PICS bags for the storage of sorghum grains over a 12-months period, also showed that *R. dominica*, a primary pest of stored sorghum was able to sustain its population during the storage period. In conclusion PICS bags can be recommended for safely storing sorghum grains over a 12-months period but investigations should be carried out to understand survival of *R. dominica* immature stages under low-oxygen conditions.
